# Systemic Therapy for Oligoprogression in Patients with Metastatic NSCLC Harboring Activating EGFR Mutations

**DOI:** 10.3390/cancers14030832

**Published:** 2022-02-06

**Authors:** Antonio Rossi, Domenico Galetta

**Affiliations:** 1 Oncology Centre of Excellence, Therapeutic Science & Strategy Unit, IQVIA, 20124 Milan, Italy; 2 Medical Thoracic Oncology Unit, IRCCS Istituto Tumori “Giovanni Paolo II”, 70124 Bari, Italy; galetta@teseo.it

**Keywords:** chemotherapy, EGFR, mutations, NSCLC, oligoprogression, oncogene-addicted, targeted therapy, TKIs

## Abstract

**Simple Summary:**

The oligoprogression concept is characterized by a limited number and/or sites of metastases in which a disease progression appears, and a more indolent tumor biology, raised specifically for oncogene addicted non-small cell lung cancer (NSCLC), including the epidermal growth factor receptor (*EGFR*)-mutant group. The optimal approach to the diagnosis and management of this disease state is not yet established. In fact, significant gaps still exist in our understanding of patient selection, type of progression, mechanisms responsible of intrinsic and acquired resistance, optimal systemic therapy, and the tumor microenvironment. Some therapeutic approaches are investigated and discussed in this review. Well-designed prospective clinical trials need to facilitate the development of therapeutic strategies beyond progression after first-line EGFR-inhibitor treatment failure.

**Abstract:**

After a variable period of activity of the epidermal growth factor receptor tyrosine kinase inhibitor (EGFR-TKI) treatment, patients with advanced non-small cell lung cancer (NSCLC) harboring *EGFR* mutations develop resistance to these TKIs. In some cases, an oligoprogression is diagnosed, and its management is still controversial. The oligoprogression represents an intermediate stage of metastatic NSCLC between localized and widely disseminated disease, and is characterized by a limited number and/or sites of metastases in which a disease progression appears, together with a more indolent tumor biology. Currently, the management of oligoprogressed NSCLC involves local treatment, including radiotherapy and/or surgery, to control the progressive lesions. Systemic therapy should also be a potential approach to boost the activity of EGFR-TKIs. However, considering the lack of large trials addressing this topic, the optimal therapeutic strategies remain undefined and should be evaluated on an individualized basis. In this paper, we review the most relevant scientific evidence of continuing the systemic therapy with the same EGFR-TKI for the management of patients with NSCLC harboring EGFR mutations and oligoprogressed to first-line EGFR-TKIs, also discussing the controversies and potential future directions.

## 1. Introduction

The management of advanced stage non-small cell lung cancer (NSCLC) has been revolutionized by the advent of targetable biomarkers. Among these, predictive biomarkers, such as the epidermal growth factor receptor (*EGFR*) mutations, the anaplastic lymphoma kinase (*ALK*) and proto-oncogene tyrosine-protein kinase 1 (*ROS1*) rearrangements, B-RAF proto-oncogene serine/threonine kinase (*BRAF*) V600E mutations, are included. The oncogene-addicted group, representing around 25–30% of metastatic NSCLC patients of prevalently non-squamous histology, can be treated with the corresponding oral small molecules tyrosine kinase inhibitors (TKIs) [1,2]. However, after an initial activity of first-line TKIs treatment, lasting around 10–14 months, a disease progression was reported in the majority of patients with the mechanisms of resistance that may vary. From a clinical point of view, in this subgroup, we can identify at least three distinct patterns of progression: isolated central nervous system progression, oligoprogression, and widespread systemic progression [3].

Approximately 20–30% of NSCLC patients with *EGFR* mutations who received EGFR-TKIs were non-responsive or responded only for a short time due to intrinsic resistance to EGFR-TKIs. In most cases, the progression is due to the onset of acquired resistance to the corresponding TKIs with new genetic alterations arising in the *EGFR* pathway or other cross-talk pathways, and for which new corresponding inhibitors are already available in early- and late-stage clinical trials [4]. 

In this article, we review the most relevant scientific evidence of continuing the systemic therapy with the same EGFR-TKI, with or without other agents, for the management of patients with NSCLC harboring *EGFR* mutations and oligoprogressed to first-line EGFR-TKIs, also discussing the controversies and potential future directions. 

## 2. Oligoprogressive Disease

The oligoprogression represents an intermediate stage of metastatic NSCLC between localized and widely disseminated disease, and is a relatively new concept that emerged when more effective systemic therapies became available. The consensus definition of oligoprogression is that it is characterized by both a limited number and/or sites of metastases, in which a disease progression appears, typically limited to patients with up to 5 progressive lesions, and a more indolent tumor biology [5]. Several concomitant parameters can modulate the anatomical pattern of treatment failure, such as the molecular evolution of cancer cells and changes in the tumor microenvironment. Oligoprogression is common in oncogene-addicted NSCLC treated with TKIs and developing an acquired resistance representing a model disease for the study of this condition. An appropriate management of oligoprogression has resulted in a median time-to-next-treatment gain and substantial overall survival (OS) improvement [5].

Moreover, this clinical evaluation has been partially incorporated in the new TNM classification. In fact, classically, all metastatic NSCLC patients have been grouped in a unique category under the M descriptor (stage IV). In the eighth TNM classification of lung cancer, the M descriptor has been subdivided into three categories: M1a, grouping cases with separate tumor nodule(s) in a contralateral lung lobe, pleural nodules or malignant pleural or pericardial effusion; M1b, grouping cases with single extrathoracic metastasis or involvement of a single distant (non-regional) node; and M1c, including cases with multiple extrathoracic metastases in one or several organs [6]. Considering that about 70% of all new NSCLC diagnosis have advanced disease, it is striking that they may be classified by only three categories according to prognosis. However, this can be considered as the first step to providing a definition for an oligometastatic disease stage in NSCLC to be considered also for defining the oligoprogression.

The hypothesis that the resistant clone progresses clinically, but the non-progressing clone may still be sensitive to TKIs, has led to use a local therapy of progressing lesions to eradicate the resistant clones and allow for the continuation of TKI to control the sensitive clones. Overall, the management of oligoprogression is controversial due to the limited clinical data, and involves mainly radical local treatment, such as radiotherapy or surgery, to achieve disease control.

In many cases, in the presence of a progression, including the oligoprogression, the current line of systemic treatment is discontinued, and a switch to a salvage line of systemic therapy is considered. The alternative strategy for oligoprogression is to continue the systemic treatment beyond progression. The choice varies depending on a number of patient factors, the NSCLC characteristics, and the number of subsequent lines of systemic treatments available. In NSCLC patients with *EGFR* mutations, a rebound tumor flare, characterized by a sudden marked increase in tumor growth, with the worsening of disease-related symptoms, and increased radiological activity on positron emission tomography/computed tomography (PET-CT) scan, which can occur with the interruption of TKI in about 20% of the patients, may play a role in this choice [7].

Different strategies have been pursued for continuing first-line EGFR-TKIs in patients with *EGFR* mutated NSCLC after the development of disease oligoprogression, such as continuation on EGFR-TKI alone or with chemotherapy, or with another targeted agent. 

## 3. Continuing EGFR-TKI Alone

In patients experiencing an oligoprogression after a durable response to EGFR-TKI, the strategy to maintain the inhibition of *EGFR* inhibitor-sensitive clones, to avoid the potential subsequent rebound tumor flare and symptomatic progression, is frequently used in clinical practice. This goal can be reached through the continuation beyond the oligoprogression of the same EGFR-TKI, in order to continue the control of the disease, including the sites of progression. This approach was evaluated mainly in retrospective analyses (Table 1).

NSCLC Japanese patients harboring activating *EGFR* mutations and radiologically progressed on EGFR-TKI treatment as the first- or second-line setting, were classified into two groups: 39 patients received continuous EGFR-TKI and 25 patients switched to chemotherapy. The median OS was 32.2 months in the patients continuing EGFR-TKI, and 23.0 months in those switching to cytotoxic chemotherapy. In the patients receiving first-line EGFR-TKI treatment, 18 in the continuous group and 9 in the switching group, the median progression-free survival (PFS) was 14.4 and 12.4 months, respectively [8].

Another Japanese retrospective analysis included 134 NSCLC patients harboring *EGFR* mutations and received gefitinib as first- or second-line treatment. The median duration of continued gefitinib therapy beyond progression was 3.2 months with a median OS of 14.3 months [9].

In a further analysis, despite RECIST defined progression, 49 patients out of 56 who continued on gefitinib or erlotinib beyond progression, the median duration of treatment was 10.1 months. The median time-to-progression (TTP) was 10.8 months in the 49 patients who continued TKI versus 9.2 months in 7 patients who discontinued TKI at progression. However, 15 out of 49 (31%) patients who continued TKI received additional systemic anti-cancer therapeutic agents while on TKI and mainly chemotherapy-based treatments [10].

Interestingly, in these analyses, patients who discontinued TKI at progression and receiving chemotherapy, were significantly younger compared to those who continued TKI beyond progression. The reason might be the feeling of investigators who preferred to change therapy in younger patients avoiding the risk of a further burden progression of disease with a worsening of general conditions that should preclude any other therapies.

A prospective phase II study, the ASPIRATION trial, investigated the efficacy of first-line erlotinib therapy in patients with NSCLC with activating *EGFR* mutations and post-progression erlotinib therapy. A total of 207 patients were enrolled, with 171 progressive disease events diagnosed while on erlotinib. Of these patients, clinicians chose to continue erlotinib in 93 (54%) patients beyond progression. The primary endpoint was PFS1, while for patients who were allowed to continue erlotinib therapy post-progression until the investigator’s decision, PFS2 was also evaluated. Moreover, an important outcome relevant to clinical practice was the extension of PFS with treatment beyond progression (the difference between PFS1 and PFS2, which was conducted as a post hoc analysis). The median PFS1 was 10.8 months for the 207 patients, while the median PFS2 was 14.1 months for the 93 patients who continued erlotinib therapy following progression. Post hoc exploratory analyses showed that the median PFS1 of the 93 patients who had treatment beyond PD was 11.0 months, with a difference between PFS1 and PFS2 of 3.1 months [11]. 

## 4. Continuing EGFR-TKI Plus Chemotherapy

The addition of cytotoxic chemotherapy to the continuation of EGFR-TKI may be a more effective antitumor treatment strategy to overcome molecular mechanisms of acquired resistance for selected patients.

A retrospective study analyzed 78 *EGFR* mutant NSCLC patients who developed an acquired drug resistance, according to the Jackman criteria [12]. These patients were treated at progression with either continuing erlotinib plus chemotherapy or chemotherapy alone. In the group treated with erlotinib plus chemotherapy, the objective response rate (ORR) was 41% versus 18% reported by the chemotherapy group. Although no significant survival improvement was observed, these data suggest better clinical activity with the addition of chemotherapy to continuing erlotinib [13]. 

The prospective IMPRESS trial addressing this question showed that the continuation of gefitinib with platinum-based chemotherapy versus chemotherapy alone, after radiological disease progression on first-line gefitinib, did not prolong PFS in patients. In fact, 265 patients were randomized to receive cisplatin/pemetrexed regimen plus gefitinib or placebo. The median PFS was 5.4 months in both groups. No differences between the treatment groups for the other outcomes were reported, including the quality of life [14].

In the IMPRESS trial, the T790M status tested on plasma circulating free tumor-derived DNA using a quantitative emulsion (BEAMing) digital PCR assay (Sysmex), conducted at a central laboratory (positivity defined as 0.02% mutant DNA fraction), was available for 247 (93.2%) patients. The median PFS for the T790M mutation-positive subgroup was 4.6 versus 5.3 months for the gefitinib plus chemotherapy and placebo plus chemotherapy groups, respectively. The median PFS for the T790M mutation-negative subgroup was 6.7 versus 5.4 months, respectively [15]. These interesting results firstly suggest that, following acquired resistance to first-line gefitinib, there appear to be two distinct patient populations defined by the T790M genotype, regarding first-line first- and second-generation EGFR-TKIs. For plasma T790M-positive patients, gefitinib should not be continued, while, for plasma T790M-negative patients, the continuation of gefitinib in combination with platinum-based doublet chemotherapy may potentially offer some clinical benefits (Table 2).

A deeper knowledge of the mechanisms of resistance to EGFR-TKIs therapy and the development of osimertinib, third-generation EGFR-TKI, led to the definition of the strategic approach for T790M-positive patients [16]. For other mechanisms of resistance, for which no specific therapeutic strategy is available, the combination of chemotherapy plus continuing first-line EGFR-TKI might be a potential strategic approach, but this requires further confirmation in a prospective randomized placebo-controlled study. 

## 5. Continuing EGFR-TKI Plus Other Targeted Agents

The continuing improvements in the knowledge of the acquired EGFR-independent mechanisms of resistance to EGFR-TKIs, including oncogenic gene fusions, gene amplification, mutations affecting gene encoding for cell-cycle proteins, the activation of the epithelial-to-mesenchymal transition (EMT), as well as the NSCLC to SCLC histologic transformation, led to the investigation of the combination of additional targeted agents against identified secondary resistance to the previous EGFR-TKI, to bypass pathways or genetic aberrations [17]. The FLOWER observational multicenter study, performed on patients with *EGFR*-mutant advanced NSCLC receiving first-line osimertinib, investigated the type and pattern of disease progression with osimertinib. In this study, progressive disease (PD) was reported in 34.9% of patients (*n* = 44/126), of whom 18.2% (*n* = 8/44) had isolated PD, considered as the appearance or growth of 1 lesion; 20.5% (*n* = 9/44) had oligoprogression, considered as the onset of ≤3 lesions in 2 organs; and 54.5% (*n* = 24/44) had systemic progression, evaluated as the appearance or progression in >3 lesions, and in 6.8% (*n* = 3/44) of the cases an unknown type of progression was reported. The most frequent PD sites were the lung, bone and brain [18].

Combination regimens investigated EGFR-TKIs with other targeted agents reporting, in some cases, disappointing results [7], but also the interesting strategies and data that are discussed here.

The mesenchymal–epithelial transition receptor (*MET*) gene amplification is detected in approximately 5–22% of NSCLC patients who progress on first-line treatment EGFR-TKIs, and is associated with poor prognosis. It leads to the *EGFR*-independent phosphorylation of the ErbB3 receptor, resulting in the subsequent bypass activation of the PI3K/Akt/mTOR pathway. *MET* gene amplification is the most common cause of bypass pathway activation involved in acquired resistance to EGFR-TKIs [19,20]. Preclinical results show the efficacy of combined MET-EGFR inhibition in overcoming resistance to EGFR-TKIs. This is a potential treatment strategy for *EGFR*-mutant advanced/metastatic NSCLC patients who progress to EGFR-TKI treatment due to the acquisition of MET aberrations [21]. Most trials evaluated the combination of an EGFR-TKI, which was not always used in the previous treatment, plus a MET inhibitor, savolitinib, capmatinib or tepotinib. Considering that it was not possible to extrapolate the data concerning the continuation of the first-line EGFR-TKI with the addition of the MET inhibitor, these studies are beyond the scope of this review [22,23,24,25,26,27,28].

The phase II ongoing ORCHARD open label, multicenter, biomarker-directed, platform study is evaluating the resistance mechanisms of first-line osimertinib identifying optimal post-progression therapies. The interim analysis reported the preliminary results concerning the administration of osimertinib plus savolitinib in *EGFR*-mutant NSCLC and *MET* amplification. The investigators reported an ORR, the primary endpoint in the first 17 evaluable patients, of 41%. Grade ≥ 3 adverse events, most commonly pneumonia and a decreased neutrophil count, and serious adverse events were reported in 30% each. A total of 3 patients (15%) discontinued combination treatment due to adverse events (Table 3) [29]. A further exploration of this combination is underway in the SAVANNAH phase II study. Eligible patients will have histologically/cytologically confirmed *EGFR*-mutant NSCLC, and *MET* amplification disease by central fluorescence in situ hybridization (FISH), central immunohistochemistry (IHC), or local next generation sequencing (NGS—retrospectively confirmed by central FISH/IHC). The primary objective is ORR [30].

The combination of osimertinib plus tepotinib, an oral, highly selective, potent MET tyrosine kinase inhibitor, is being assessed in the ongoing INSIGHT2 international, open-label, multicenter phase II trial in patients with advanced/metastatic *EGFR*-mutant NSCLC and acquired resistance to first-line osimertinib, and *MET* amplification determined centrally by FISH (gene copy number ≥ 5 and/or MET/CEP7 ≥ 2) at the time of progression. The primary end point is ORR [31]. 

## 6. Central Nervous System Oligoprogression

Central nervous system (CNS) progression was estimated to be the first site of recurrence in about 33% of cases, independent of disease control elsewhere, in *EGFR*-mutated NSCLC patients who respond to EGFR-TKI therapy. Studies have demonstrated lower cerebrospinal fluid (CSF) concentrations of EGFR-TKIs compared to plasma concentrations, leading to consider the inadequate penetration of EGFR-TKI into the CSF, as one of the potential resistance mechanisms responsible for CNS disease progression [32,33].

In the presence of CNS metastases, the treatment decisions depend on the degree of progression both intracranially and extracranially, and on the systemic treatment options remaining for the patient. Symptomatic CNS metastases require corticosteroid treatment and a referral to radiation oncology and/or neurosurgery, regardless of the presence of a driver alteration. In asymptomatic CNS progression in oncogene-driven NSCLC, in the context of widespread progression, a change of systemic therapy should be considered and, in particular, the availability of another targeted agent with CNS activity should be prioritized. In patients with CNS oligoprogression alone, either the continuation of the current targeted therapy plus local brain treatment, or another targeted agent with proven CNS activity to withhold local brain therapy, and switching to another targeted agent, with careful brain surveillance, could also be considered [34].

In this context, the third-generation EGFR-TKIs, osimertinib and almonertinib, showed remarkable activity against brain metastases. The AURA3 trial randomized *EGFR*-mutant NSCLC patients who progressed to first-generation EGFR-TKIs, due to the acquired EGFR-T790M mutation, to receive osimertinib or platinum-pemetrexed chemotherapy. In the subgroup of patients with CNS metastases, osimertinib improved the median CNS PFS with respect to the chemotherapy group (11.7 vs. 5.6 months, respectively). These results were reported regardless of prior brain radiotherapy [35]. Similarly, the FLAURA study, comparing first-line osimertinib versus first-generation EGFR-TKIs, gefitinib or erlotinib, confirmed that osimertinib reduced the risk of CNS progression (20% vs. 39%, respectively), as well as the onset of new brain metastases (12% vs. 30%, respectively). These further data highlight the protective role of osimertinib in the control of CNS lesions [36].

Almonertinib is a novel orally available, pyrimidine-based, third-generation EGFR-TKI with high selectivity and potent inhibitory activity against both *EGFR*-sensitizing and *EGFR*-T790M mutations. In the APOLLO study, patients who developed an *EGFR*-T790M mutation after progression to first- or second-generation EGFR-TKIs, received almonertinib. The median PFS in patients with CNS lesions was 10.8 months with a CNS ORR of 60.9% [37]. Almonertinib is being investigated as first-line therapy for *EGFR*-mutant NSCLC patients with CNS metastases, in the phase II ACHIEVE trial (NCT04808752).

## 7. Discussion

The disease progression with EGFR-TKIs shows distinct patterns of growth: isolated central nervous system progression, oligoprogression or widespread systemic progression [3], upon which the therapeutic decisions should be taken. Oligoprogression is considered in a situation in which an *EGFR*-mutant advanced NSCLC patient develops disease progression in one or a limited number of sites after an EGFR-TKI period of stable disease or an ORR. The consensus definition of oligoprogression describes up to 5 progressive lesions and can be estimated with a frequency ranging from 15% to 47% during EGFR-TKI treatment. Oligoprogression is related to tumor heterogeneity, and the development of an isolated resistant subclone at only 1 or few metastatic site(s) [5,34].

A common approach for treating oligoprogression in *EGFR*-mutant advanced NSCLC patients is to continue the EGFR-TKI that is controlling the greater proportion of the disease, while using local ablative therapy to eradicate the resistant clones in the area or areas of progression. This therapeutic approach is supported by several, mainly retrospective, case series [34]. 

In this article, we reviewed the available data for continuing first-line EGFR-TKI in patients with *EGFR*-mutant NSCLC after the development of disease progression. The retrospective and prospective data show that continuing first-line EGFR-TKI over progression alone, or in combination with other systemic treatment, such as chemotherapy or other targeted agents, seems useful at least in a well-defined population. In those for which the progression is driven by the onset of a new molecular alteration, the combination of continuing first-line EGFR-TKI and the addition of the specific inhibitor is under investigation, in particular, the combination of osimertinib and MET inhibitor. The population who may gain a greater benefit from the continuation of first-line EGFR-TKI plus chemotherapy is represented by the patients in which a specific molecular alteration responsible for the progression is not targetable or is not found. All these considerations are relevant, providing that the patient has progressed without a bulky disease and with a good general clinical condition without severe symptoms. In this regard, we have to also consider that by stopping the EGFR-TKI treatment due to the progression of the disease, there is a risk of a rebound tumor flare, which can occur in about 20% of the patients, and may play a role in treatment choice.

A limitation of the results available is that the studies do not report the burden of disease progression, with no specification if the patients enrolled are oligo or widespread progressors. However, both these studies showed a delay in switching to a salvage line of systemic therapy and avoiding the potential risk of flares.

A portion of *EGFR*-mutant NSCLC patients, treated with the third-generation of EGFR-TKIs, progresses due to the onset, as an acquired mechanism of resistance, of a tertiary *EGFR*-C797S mutation. A novel class of allosteric fourth generation EGFR-TKIs is in an advanced stage of development, with the intent to overcome acquired resistance to third-generation EGFR-TKIs. These drugs targeting the allosteric site of the EGFR receptor which, being situated away from the ATP binding site commonly targeted by the other EGFR-TKIs, is not affected by the *EGFR*-T790M and *EGFR*-C797S mutations [38,39].

Among the fourth generation EGFR-TKIs, EAI045 is the first allosteric EGFR inhibitor designed to target drug resistant *EGFR*-T790M and *EGFR*-C797S mutants [40]. JBJ-04-125-02 is another allosteric EGFR-TKI appearing to be more potent than EAI045 [41]. Preclinical in vitro and in vivo data are very interesting and promising for their investigation in early-stage clinical development. However, both EAI045 and JBJ-04-125-02 cannot overcome the resistance mediated by the Ex19del/T790M/C797S triple mutant. On the other hand, CH7233163, TQB3804 and BBT-176 are novel fourth-generation EGFR-TKIs, which are capable of overcoming the resistance mediated by the Ex19del/T790M/C797S and L858R/T790M/C797S triple mutation. BLU-945, another fourth-generation EGFR-TKI, achieved in vitro robust inhibition of Ex19del/T790M/C797S and L858R/T790M/C797S triple mutation, rather than the *EGFR* wild-type, as well [42].

This continuing increase in knowledge of the genomic profiling of patient tissue at greater speeds and at lower costs allowed for the development of NGS platforms and targeted gene panels. The therapeutic strategy to use for *EGFR*-mutant advanced NSCLC patients progressed to first-line EGFR-TKI may use NGS analysis to detect early signals of drug resistance, to potentially direct the switching to an alternative antitumor drug or combination to overcome relevant subclonal progression. In this context and considering that in lung cancer the tumor is often difficult to reach and may need an invasive and potentially harmful procedure, the continuing development of the analyses of liquid biopsies can noninvasively detect any targetable genomic alteration and guide corresponding targeted therapy. Moreover, liquid biopsy might help in monitoring the response to treatment and exploring the genetic changes at resistance, overcoming spatial and temporal heterogeneity [43,44].

## 8. Conclusions

The understanding and treatment of metastatic NSCLC has seen significant advances within the last few years. Oncogene-addicted NSCLC includes several subgroups of patients who can greatly benefit from specific inhibitors. The continuous research and development of new generation inhibitors is changing this scenario. However, significant gaps still exist in our understanding of patient selection, type of progression, mechanisms responsible of intrinsic and acquired resistance, optimal systemic therapy, and the tumor microenvironment.

**Figure 1:**
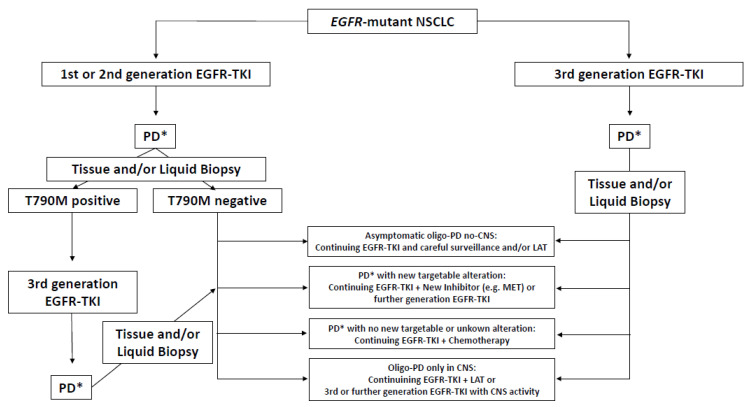
Suggested potential algorithm for continuing first-line EGFR-TKI after oligoprogression in EGFR-mutant NSCLC patients (to consider according to the local regulatory agency). * Oligoprogression or not clinically significant widespread progression; PD: progression disease; EGFR-TKI: epidermal growth factor receptor tyrosine kinase inhibitor; NSCLC: non-small-cell lung cancer; CNS: central nervous system; LAT: local ablative therapy; and MET: mesenchymal-epithelial transition receptor.

Oligoprogression is a recently acknowledged pattern of disease progression on systemic anti-cancer therapy raised specifically for oncogene addicted NSCLC, including the *EGFR*-mutant group. The optimal approach to the diagnosis and management of this disease state has not yet been established. The pattern of progression and time to EGFR-TKI response are relevant factors to be taken into account at the time of progression, in order to select the patients who are candidates for a therapeutic switch or rechallenge. Some approaches were investigated and discussed in this review, and can be summarized by Figure 1. However, the validation of these results is required with well-designed prospective clinical trials to facilitate the development of therapeutic strategies beyond progression after first-line EGFR-TKI treatment failure.

## Figures and Tables

**Table 1:** Results of the main studies evaluating the continuing EGFR-TKI after progression in *EGFR*-mutant NSCLC patients.

Study, Year	Region	Study	Prior EGFR-TKI	Prior Line of EGFR-TKI	No.Pts: Therapy	Median Age (Years)	ORR (%)	PFS (Months)	OS (Months)	Reference
Nishie et al., 2012	Japan	Retrospective	Not specified	First: 27 pts	39: EGFR-TKI	69	NR	14.4 *	32.2	[8]
				Second: 37 pts	25: CT/Other	58		12.4 *	23.0	
Asami et al., 2013	Japan	Retrospective	Gefitinib	First: 51 pts	134: Gefitinib	<75: 84 pts	NR	9.5	NR	[9]
				Second: 83 pts		≥75: 50 pts				
Nishino et al., 2013	U.S.	Retrospective	Erlotinib: 50 pts	First: 56 pts	49: EGFR-TKI	64	NR	10.8 °	NR	[10]
			Gefitinib: 6 pts		7: CT/Other	48		9.2 °		
ASPIRATION, 2016	Asia	Prospective	Erlotinib	First: 207 pts	93: Erlotinib	60.8	66.2	14.1	33.6	[11]
					78: No erlotinib			7.4	22.5	

* In the patients receiving first-line EGFR-TKI treatment; ° TTP: time to progression; EGFR-TKI: epidermal growth factor receptor-tyrosine kinase inhibitor; NSCLC: non-small-cell lung cancer; No.pts: number of patients; ORR: objective response rate; PFS: progression free survival; OS: overall survival; CT: chemotherapy; and NR: not reported; U.S.: United States of America.

**Table 2:** Results of the main studies evaluating the continuing EGFR-TKI plus chemotherapy after progression in *EGFR*-mutant NSCLC patients.

Study, Year	Region	Study	Prior EGFR-TKI	Prior Line of EGFR-TKI	No.Pts: Therapy	Median Age (Years)	ORR (%)	PFS (Months)	OS (Months)	Reference
Goldberg et al., 2013	U.S.	Retrospective	Erlotinib: 62 pts	First: 57 pts	34: Erlotinib+CT 44: CT	58	41	4.4	14.2	[13]
			Gefitinib/other: 16 pts	Second: 21 pts		58	18	4.2	15.0	
IMPRESS, 2015, 2017	E.U./Asia	Prospective	Gefitinib	First	133: Gefitinib+CT vs	60	32	5.4	13.4	[14,15]
					132: Placebo+CT	58	34	5.4	19.5	
IMPRESS, 2017	E.U./Asia	Prospective	Gefitinib	First: T790M+ 142 pts	81: Gefitinib+CT vs	57.8	28.4	4.6	10.8	[15]
					61: Placebo+CT	55.8	39.5	5.3	14.1	
				First: T790M- 105 pts	46: Gefitinib+CT vs	55.8	36.8	6.7	21.4	
					59: Placebo+CT	58.5	32.3	5.4	22.5	

EGFR-TKI: epidermal growth factor receptor-tyrosine kinase inhibitor; NSCLC: non-small-cell lung cancer; No.pts: number of patients; ORR: objective response rate; PFS: progression free survival; OS: overall survival; and CT: chemotherapy; U.S.: United States of America; E.U.: European Union.

**Table 3:** Results of the main studies evaluating the continuing first-line EGFR-TKI plus other targeted agents after progression in *EGFR*-mutant NSCLC patients.

Study, Year	Region	Study	Prior EGFR-TKI	Mechanisms of Resistance	No.Pts: Therapy	ORR (%)	PFS (Months)	OS (Months)	Reference
Yu et al., 2021	U.S.	Prospective	Osimertinib	*MET* amplification	17: Osimertinib+Savolitinib	41	NR	NR	[29]

EGFR-TKI: epidermal growth factor receptor-tyrosine kinase inhibitor; MET: mesenchymal–epithelial transition receptor; NSCLC: non-small-cell lung cancer; No.pts: number of patients; ORR: objective response rate; PFS: progression free survival; OS: overall survival; and NR: not reported; U.S.: United States of America.

## References

[1] Planchard D., Popat S., Kerr K., Novello S., Smit E.F., Faivre-Finn C., Mok T.S., Reck M., Van Schil P.E., Hellmann M.D. (2018). Metastatic non-small-cell lung cancer: ESMO Clinical Practice Guidelines for diagnosis, treatment and follow-up. Ann. Oncol..

[2] Hanna N., Robinson A.G., Temin S., Baker S., Brahmer J.R., Ellis P.M., Gaspar L.E., Haddad R.Y., Hesketh P.J., Jain D. (2021). Therapy for stage IV non-small-cell lung cancer with driver alterations: ASCO and OH (CCO) joint guideline update. J. Clin. Oncol..

[3] Gandara D.R., Li T., Lara P.N., Kelly K., Riess J.W., Redman M.W., Mack P.C. (2014). Acquired resistance to targeted therapies against oncogene-driven non-small-cell lung cancer: Approach to subtyping progressive disease and clinical implications. Clin. Lung Cancer.

[4] Malapelle U., Muscarella L.A., Pisapia P., Rossi A. (2020). Targeting emerging molecular alterations in the treatment of non-small cell lung cancer: Current challenges and the way forward. Expert Opin. Investig. Drugs.

[5] Patel P.H., Palma D., McDonald F., Tree A.C. (2019). The dandelion dilemma revisited for oligoprogression: Treat the whole lawn or weed selectively?. Clin. Oncol..

[6] Rami-Porta R., Asamura H., Travis W.D., Rusch V.W. (2017). Lung cancer-major changes in the American Joint Committee on Cancer eight edition staging manual. CA Cancer J. Clin..

[7] Yap T.A., Macklin-Doherty A., Popat S. (2017). Continuing EGFR inhibition beyond progression in advanced non-small cell lung cancer. Eur. J. Cancer.

[8] Nishie K., Kawaguchi T., Tamiya A., Mimori T., Takeuchi N., Matsuda Y., Omachi N., Asami K., Okishio K., Atagi S. (2012). Epidermal growth factor receptor tyrosine kinase inhibitors beyond progressive disease. A retrospective analysis for Japanese patients with activating EGFR mutations. J. Thorac. Oncol..

[9] Asami K., Okumab T., Hirashimac T., Kawaharad M., Atagia S., Kawaguchia T., Okishioa K., Omachia N., Takeuchia N. (2013). Continued treatment with gefitinib beyond progressive disease benefits patients with activating EGFR mutations. Lung Cancer.

[10] Nishino M., Cardarella S., Dahlberg S.E., Jackman D.M., Ramaiya N.H., Hatabu H., Rabin M.S., Jänne P.A., Johnson B.E. (2013). Radiographic assessment and therapeutic decisions at RECIST progression in EGFR-mutant NSCLC treated with EGFR tyrosine kinase inhibitors. Lung Cancer.

[11] Park K., Yu C.J., Kim S.W., Lin M.C., Sriuranpong V., Tsai C.M., Lee J.S., Kang J.H., Chan K.C.A., Perez-Moreno P. (2016). First-line erlotinib therapy until and beyond response evaluation criteria in solid tumors progression in Asian patients with epidermal growth factor receptor mutation-positive non-small-cell lung cancer. The ASPIRATION study. JAMA Oncol..

[12] Jackman D., Pao W., Riely G.J., Engelman J.A., Kris M.G., Jänne P.A., Lynch T., Johnson B.E., Miller V.A. (2010). Clinical definition of acquired resistance to epidermal growth factor receptor tyrosine kinase inhibitors in non-small-cell lung cancer. J. Clin. Oncol..

[13] Goldberg S.B., Oxnard G.R., Digumarthy S., Muzikansky A., Jackman D.M., Lennes I.T., Sequist L.V. (2013). Chemotherapy with erlotinib or chemotherapy alone in advanced non-small cell lung cancer with acquired resistance to EGFR tyrosine kinase inhibitors. Oncologist.

[14] Soria J.C., Wu Y.L., Nakagawa K., Kim S.W., Yang J.J., Ahn M.J., Wang J., Yang J.C.H., Lu Y., Atagi S. (2015). Gefitinib plus chemotherapy versus placebo plus chemotherapy in EGFR-mutation-positive non-small-cell lung cancer after progression on first-line gefitinib (IMPRESS): A phase 3 randomised trial. Lancet Oncol..

[15] Mok T.S.K., Kim S.W., Wu Y.L., Nakagawa K., Yang J.J., Ahn M.J., Wang J., Yang J.C.H., Lu Y., Atagi S. (2017). Gefitinib plus chemotherapy versus chemotherapy in epidermal growth factor receptor mutation-positive non-small-cell lung cancer resistant to first-line gefitinib (IMPRESS): Overall survival and biomarker analyses. J. Clin. Oncol..

[16] Mok T.S., Wu Y.-L., Ahn M.-J., Garassino M.C., Kim H.R., Ramalingam S.S., Shepherd F.A., He Y., Akamatsu H., Theelen W.S.M.E. (2017). Osimertinib or platinum-pemetrexed in EGFR T790M–positive lung cancer. N. Engl. J. Med..

[17] Papini F., Sundaresan J., Leonetti A., Tiseo M., Rolfo C., Peters G.J., Giovannetti E. (2021). Hype or hope Can combination therapies with third-generation EGFR-TKIs help overcome acquired resistance and improve outcomes in EGFR-mutant advanced/metastatic NSCLC?. Crit. Rev. Oncol. Hematol..

[18] Lorenzi M., Ferro A., Cecere F., Scattolin D., Del Conte A., Follador A., Pilotto S., Polo V., Santarpia M., Chiari R. (2021). First-line osimertinib in patients with EGFR-mutant advanced non-small cell lung cancer: Outcome and safety in the real world: FLOWER study. Oncologist.

[19] Zhang Z., Yang S., Wang Q. (2019). Impact of MET alterations on targeted therapy with EGFR-tyrosine kinase inhibitors for EGFR-mutant lung cancer. Biomark. Res..

[20] Ramalingam S.S., Cheng Y., Zhou C., Ohe Y., Imamura F., Cho B.C., Lin M.C., Majem M., Shah R., Rukazenkov Y. (2018). Mechanisms of acquired resistance to first-line osimertinib: Preliminary data from the phase III FLAURA study. Ann. Oncol..

[21] Friese-Hamim M., Bladt F., Locatelli G., Stammberger U., Blaukat A. (2017). The selective c-Met inhibitor tepotinib can overcome epidermal growth factor receptor inhibitor resistance mediated by aberrant c-Met activation in NSCLC models. Am. J. Cancer Res..

[22] Sequist L.V., Han J.Y., Ahn M.J., Cho B.C., Yu H., Kim S.W., Yang J.C., Lee J.S., Su W.C., Kowalski D. (2020). Osimertinib plus savolitinib in patients with EGFR mutation-positive, MET-amplified, non-small-cell lung cancer after progression on EGFR tyrosine kinase inhibitors: Interim results from a multicentre, open-label, phase 1b study. Lancet Oncol..

[23] Oxnard G.R., Yang J.C., Yu H., Kim S.W., Saka H., Horn L., Goto K., Ohe Y., Mann H., Thress K.S. (2020). TATTON: A multi-arm, phase Ib trial of osimertinib combined with selumetinib, savolitinib, or durvalumab in EGFR-mutant lung cancer. Ann. Oncol..

[24] Yang J.J., Fang J., Shu Y.Q., Chang J.H., Chen G.Y., He J.X., Li W., Liu X.Q., Yang N., Zhou C. (2021). A phase Ib study of the highly selective MET-TKI savolitinib plus gefitinib in patients with EGFR-mutated, MET-amplified advanced non-small-cell lung cancer. Investig. New Drugs.

[25] Han J., Sequist L., Ahn M., Cho B.C., Yu H., Kim S., Yang J.C., Lee J.S., Su W., Kowalski D.M. (2021). Osimertinib + savolitinib in pts with EGFRm MET-amplified/overexpressed NSCLC: Phase Ib TATTON parts B and D final analysis. J. Thorac. Oncol..

[26] Wu Y.L., Zhang L., Kim D.W., Liu X., Lee D.H., Yang J.C.H., Ahn M.J., Vansteenkiste J.F., Su W.C., Felip E. (2018). Phase Ib/II study of capmatinib (INC280) plus gefitinib after failure of epidermal growth factor receptor (EGFR) inhibitor therapy in patients with EGFR-mutated, MET factor-dysregulated non-small-cell lung cancer. J. Clin. Oncol..

[27] McCoach C.E., Yu A., Gandara D.R., Riess J.W., Vang D.P., Li T., Lara P.N., Gubens M., Lara F., Mack P.C. (2021). Phase I/II study of capmatinib plus erlotinib in patients with MET-positive non-small-cell lung cancer. JCO Precis. Oncol..

[28] Wu Y.L., Cheng Y., Zhou J., Lu S., Zhang Y., Zhao J., Kim D.W., Soo R.A., Kim S.W., Pan H. (2020). Tepotinib plus gefitinib in patients with EGFR-mutant non-small-cell lung cancer with MET overexpression or MET amplification and acquired resistance to previous EGFR inhibitor (INSIGHT study): An open-label, phase 1b/2, multicentre, randomised trial. Lancet Resp. Med..

[29] Yu H.A., Ambrose H., Baik C., Cho B.C., Cocco E., Goldberg S.B., Goldman J.W., Kraljevic S., de Langen A.J., Okamoto I. (2021). ORCHARD osimertinib + savolitinib interim analysis: A biomarker-directed phase II platform study in patients (pts) with advanced non-small cell lung cancer (NSCLC) whose disease has progressed on first-line (1L) Osimertinib. Ann. Oncol..

[30] Oxnard G.R., Cantarini M., Frewer P., Hawkins G., Peters J., Howarth P., Ahmed G.F., Sahota T., Hartmaier R., Li-Sucholeiki X. (2019). SAVANNAH: A phase II trial of osimertinib plus savolitinib for patients (pts) with EGFR-mutant, MET-driven (MET+), locally advanced or metastatic non-small cell lung cancer (NSCLC), following disease progression on osimertinib. J. Clin. Oncol..

[31] Smit E.F., Dooms C., Raskin J., Nadal E., Tho L.M., Le X., Mazieres J., Hin H.S., Morise M., Zhu V.W. (2021). INSIGHT 2: A phase II study of tepotinib plus osimertinib in MET-amplified NSCLC and first-line osimertinib resistance. Future Oncol..

[32] Heon S., Yeap B.Y., Britt G.J., Costa D.B., Rabin M.S., Jackman D.M., Johnson B.E. (2010). Development of central nervous system metastases in patients with advanced non-small cell lung cancer and somatic EGFR mutations treated with gefitinib or erlotinib. Clin. Cancer Res..

[33] Jackman D.M., Holmes A.J., Lindeman N., Wen P.Y., Kesari S., Borras A.M., Bailey C., de Jong F., Jänne P.A., Johnson B.E. (2006). Response and resistance in a non-small-cell lung cancer patient with an epidermal growth factor receptor mutation and leptomeningeal metastases treated with high-dose gefitinib. J. Clin. Oncol..

[34] Laurie S.A., Banerji S., Blais N., Brule S., Cheema P.K., Cheung P., Daaboul N., Hao D., Hirsh V., Juergens R. (2019). Canadian consensus: Oligoprogressive, pseudoprogressive, and oligometastatic non-small-cell lung cancer. Curr. Oncol..

[35] Wu Y.L., Ahn M.J., Garassino M.C., Han J.Y., Katakami N., Kim H.R., Hodge R., Kaur P., Brown A.P., Ghiorghiu D. (2018). CNS efficacy of osimertinib in patients with T790M-positive advanced non-small-cell lung cancer: Data from a randomized phase III trial (AURA3). J. Clin. Oncol..

[36] Reungwetwattana T., Nakagawa K., Cho B.C., Cobo M., Cho E.K., Bertolini A., Bohnet S., Zhou C., Lee K.H., Nogami N. (2018). CNS response to osimertinib versus standard epidermal growth factor receptor tyrosine kinase inhibitors in patients with untreated EGFR-mutated advanced non-small-cell lung cancer. J. Clin. Oncol..

[37] Lu S., Wang Q., Zhang G., Dong X., Yang C.T., Song Y., Chang G.C., Lu Y., Pan H., Chiu C.H. (2021). Efficacy of aumolertinib (HS-10296) in patients with advanced EGFR T790M+ NSCLC: Updated post-national medical products administration approval results from the APOLLO registrational trial. J. Thorac. Oncol..

[38] Leonetti A., Sharma S., Minari R., Perego P., Giovannetti E., Tiseo M. (2019). Resistance mechanisms to osimertinib in EGFR-mutated non-small cell lung cancer. Br. J. Cancer.

[39] Zhao P., Yao M.Y., Zhu S.J., Chen J.Y., Yun C.H. (2018). Crystal structure of EGFR T790M/C797S/V948R in complex with EAI045. Biochem. Biophys. Res. Commun..

[40] Wang S., Song Y., Liu D. (2017). EAI045: The fourth-generation EGFR inhibitor overcoming T790M and C797S resistance. Cancer Lett..

[41] To C., Jang J., Chen T., Park E., Mushajiang M., De Clercq D.J.H., Xu M., Wang S., Cameron M.D., Heppner D.E. (2019). Single and dual targeting of mutant EGFR with an allosteric inhibitor. Cancer Discov..

[42] Du X., Yang B., An Q., Assaraf Y.G., Cao X., Xia J. (2021). Acquired resistance to third-generation EGFR-TKIs and emerging next-generation EGFR inhibitors. Innovation.

[43] Zheng M.M., Li Y.S., Tu H.Y., Jiang B.Y., Yang J.J., Zhou Q., Xu C.R., Yang X.R., Wu Y.L. (2021). Genotyping of cerebrospinal fluid associated with osimertinib response and resistance for leptomeningeal metastases in EGFR-mutated NSCLC. J. Thorac. Oncol..

[44] Christopoulos P., Dietz S., Angeles A.K., Rheinheimer S., Kazdal D., Volckmar A.L., Janke F., Endris V., Meister M., Kriegsmann M. (2021). Earlier extracranial progression and shorter survival in ALK-rearranged lung cancer with positive liquid rebiopsies. Transl. Lung Cancer Res..

